# Supplementary material for: Evolutionary Dynamics of the Lineage 2 West Nile Virus That Caused the Largest European Epidemic: Italy 2011–2018
Source: Viruses. 2019 Sep 3;11(9):814. doi: 10.3390/v11090814 (PMC6784286; doi:10.3390/v11090814)
Supplement: Supplementary file 1 [file viruses-11-00814-s001.zip › Supplementary revised/viruses-573353-supplementary-legend.docx]

**Supplementary Materials:** Table S1: Collection years, codes, host and sampling location of the newly characterized WNV-2 Italian sequences included in the dataset; Table S2: Mean genetic distances in relation to the host.
